# High-Throughput Prediction of the Thermal and Electronic Transport Properties of Large Physical and Chemical Spaces Accelerated by Machine Learning: Charting the *ZT* of Binary Skutterudites

**DOI:** 10.1021/acsami.3c15741

**Published:** 2024-01-22

**Authors:** Julia Santana-Andreo, Antonio M. Márquez, Jose J. Plata, Ernesto J. Blancas, José-Luis González-Sánchez, Javier Fdez. Sanz, Pinku Nath

**Affiliations:** † Departamento de Química Física, Facultad de Química, Universidad de Sevilla, Seville 41012, Spain; ‡ Department of Computer Systems Engineering and Telematics, University of Extremadura, School of Technology, Cáceres 10003, Extremadura, Spain; § Institute for Chemical Reaction Design and Discovery (WPI-ICReDD), Hokkaido University, Sapporo 060-0808, Japan

**Keywords:** thermoelectrics, skutterudites, transport properties, grains, carriers, DFT

## Abstract

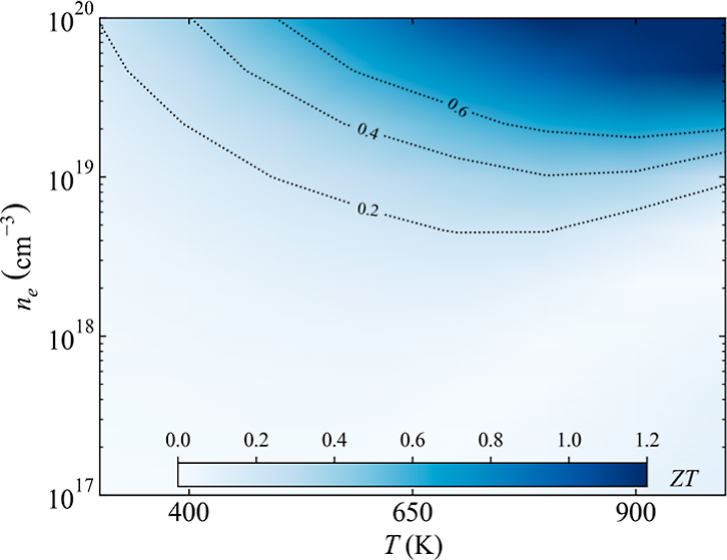

Thermal and electronic transport properties are the keys to many technological applications of materials. Thermoelectric, TE, materials can be considered a singular case in which not only one but three different transport properties are combined to describe their performance through their TE figure of merit, *ZT*. Despite the availability of high-throughput experimental techniques, synthesizing, characterizing, and measuring the properties of samples with numerous variables affecting *ZT* are not a cost- or time-efficient approach to lead this strategy. The significance of computational materials science in discovering new TE materials has been running in parallel to the development of new frameworks and methodologies to compute the electron and thermal transport properties linked to *ZT*. Nevertheless, the trade-off between computational cost and accuracy has hindered the reliable prediction of TE performance for large chemical spaces. In this work, we present for the first time the combination of new ab initio methodologies to predict transport properties with machine learning and a high-throughput framework to establish a solid foundation for the accurate prediction of thermal and electron transport properties. This strategy is applied to a whole family of materials, binary skutterudites, which are well-known as good TE candidates. Following this methodology, it is possible not only to connect *ZT* with the experimental synthetic (carrier concentration and grain size) and operando (temperature) variables but also to understand the physical and chemical phenomena that act as driving forces in the maximization of *ZT* for p-type and n-type binary skutterudites.

## Introduction

The global energy demand and its associated costs represent a challenge that transcends the fossil-fuel-renewable energy dichotomy. When approximately 70% of the energy produced by our society is wasted as heat, improving the efficiency of the grid is as important as using environmentally friendly energy sources. Thermoelectricity stands out as one of the best solutions to recycle that heat, producing electricity. Thermoelectric (TE) generators not only improve the efficiency of systems with combustion engines^1^ but have also been successfully combined with photovoltaics,^2^ biomass stoves,^3^ marine power plants,^4^ desalination devices,^5^ and wastewater plants.^6^ However, the high cost and moderate conversion efficiencies of TE materials are the main obstacles preventing their worldwide implementation.

The discovery or optimization of more efficient and less expensive TE materials cannot be seen as an easy task. During recent decades, significant resources have been dedicated to the improvement of the performance of these materials, as measured by their figure of merit, *ZT*. However, *ZT* has not been increased at the same pace over the last 70 years. During the latter half of the 20th century, the best TE materials did not surpass *ZT* values larger than one. This trend radically changed in the 21st, when materials with *ZT* values above two were reported.^7,8^ Many factors have propelled the discovery and optimization of TE materials, with computational science and atomistic simulations playing a predominant role in the TE field during the last 20 years.

The importance of computational materials science in the discovery of new TE materials has run in parallel with the development of new frameworks and methodologies to compute the electron and thermal transport properties linked to *ZT*.^9^ Predicting properties such as electrical conductivity, σ, Seebeck coefficient, *S*, or thermal conductivity, κ, stands as the most reliable approach to searching for new TE materials. A large set of methods, packages, and frameworks have been developed during the last two decades to predict the aforementioned properties.^10−14^ The main disadvantage under all of these options stems from the accuracy-computational cost trade-off. For instance, many approaches have been developed to compute the lattice thermal conductivity of crystals at very low computational cost; however, their results can only be used qualitatively or semiquantitatively.^15−17^ Accurate methods require the calculation of high-order force constants^18^ or the calculation of long ab initio molecular dynamics,^19^ which cannot be systematically used for large sets of materials. In addition to the obstacle of high computational cost, some methods require experimental data as input. For instance, the constant relaxation time approximation stands as the most extended approach^10^ for computing the electronic transport properties; however, it is usually combined with experimental electron scattering times.

In recent years, new approaches have been developed to reduce the computational cost or the need for experimental data without compromising the accuracy of the results. The use of machine learning techniques in the calculation of interatomic force constants, IFCs, has drastically reduced by up to 2 orders of magnitude the computational cost of predicting the lattice thermal conductivity of solids using the Boltzmann transport equation, BTE,^20,21^ opening the door to explore large chemical spaces.^22,23^ On the other hand, new efficient methods have been reported for calculating carrier scattering rates of semiconductors and insulators from first principles without any external parameters.^24−26^ Very recently, some authors have combined both methodologies using MLIP and AMSET packages to predict *ZT*.^27,28^ While MLIP develops moment tensor potentials to reduce the computational cost of high-order force constants, AMSET predicts the electron transport properties beyond RTA.^25^ Following this strategy, Yuan et al. explored the TE performance of CsK_2_X (X = Sb, Bi),^27^ and Bai et al. examined LaAgOX (X = S, Se). This approach not only guarantees accurate values for *ZT* but also reduces the cost, providing the opportunity to study larger sets of materials for a better understanding of the physical and chemical factors that govern thermoelectricity. In this work, the whole family of binary skutterudites MX_3_ (M = Co, Rh, Ir; X = P, As, Sb), with high potential applications in thermoelectrics, is explored. Although Co-based skutterudites are the most common subset, Rh-based and Ir-based skutterudites have also been reported as good TE candidates.^29,30^ To the best of our knowledge, we combine these new strategies to compute *ZT* for a large set of materials for the first time, highlighting the importance of extracting design rules for the discovery of new high-*ZT* skutterudites. In addition to reporting *ZT* values as a function of experimental synthetic (carrier concentration), processing (grain size), and operando (temperature) variables for this family of materials, the effects derived from the chemical composition in these binary skutterudites are evaluated.

## Methods

### Thermal Transport Properties

#### Geometry Optimization

All ground-state structures were fully relaxed (atoms and lattice parameters) with the VASP package^31,32^ using projector-augmented wave (PAW) potentials.^33^ Energies were obtained by combining the exchange–correlation functional proposed by Perdew–Burke–Ernzerhof (PBE)^34^ with Grimme D3 van der Waals corrections.^35^ Core and valence electrons were selected following the standards proposed by Calderon et al.^36^ A high-energy cutoff of 500 eV and a dense **k**-point mesh of 2050 **k**-points per reciprocal atom were used. The wave function was considered converged when the energy difference between two consecutive electronic steps was smaller than 10^–9^ eV. Geometry and lattice vectors were fully relaxed using a 32-atom conventional cell until forces over all atoms were smaller than 10^–7^ eV/Å. An additional support grid for the evaluation of the augmentation charges was included to reduce the noise in the forces.

#### Supercell Single-Point Calculations and Force Constants

IFCs were calculated using the hiPhive package, which combines the forces calculated for random atomic distortion in supercells with machine learning (ML) regression.^21^ The forces were calculated in a 2 × 2 × 2 supercell (256 atoms) using the same setup as that used for the geometry optimizations. The amplitude of the distortions applied to the atoms plays an important role in the calculation of the IFCs, so a 2-step approach was designed.^37^ First, small random distortions were generated for all the atoms of three supercells, and second- and third-order IFCs were extracted using the hiPhive package. Then, 14 new distorted supercells were created, superimposing normal modes with random phase factors and amplitudes corresponding to 300 K using the second-order IFCs obtained in the previous step. The force constants were calculated from multilinear regression to the DFT forces with the recursive feature elimination, RFE, algorithm. Although RFE is more expensive than using ordinary least-squares regression, it has been proven that this algorithm requires a smaller number of structures to converge.^38^ Additionally, reducing the number of parameters via the RFE also simplifies the model, keeping only the most relevant interaction terms. IFCs were calculated including cutoffs for second-, third-, and fourth-order terms. To ensure transferability across compounds, cutoffs were determined based on coordination shells.

#### BTE Solver

The ShengBTE code was used to calculate the lattice thermal conductivity, κ_l_, through the iterative solution of the BTE, which produces better results than the relaxation time approximation.^11^ Scattering times were computed, including isotopic and three-phonon scattering. Memory demand and the convergence of κ_l_ with the number of **q**-points were balanced using a Gaussian smearing of 0.05 and a dense mesh of 12 × 12 × 12 **q**-points. The effects of including nonanalytical contributions (NACs) on κ_l_ were tested in CoSb_3_ between 300 and 900 K, and only small changes (<2%) were found (see Supporting Information); therefore, the results reported below do not include NACs.

### Electronic Transport Properties

Electrical conductivity, the Seebeck coefficient, and the electronic contribution to thermal conductivity were calculated using the AMSET package.^25^ This code solves the Boltzmann transport equation using the Onsager coefficients to predict electronic transport properties with the wave function from a DFT calculation as the main input. Scattering rates for each temperature, doping concentration, band, and **k**-point were calculated, including scattering due to deformation potentials, polar optical phonons, and ionized impurities (see Supporting Information). Wave function coefficients were obtained using the HSE06 functional proposed by Heyd et al.^39^ using the primitive cell (16 atoms) and a dense mesh of 10 × 10 × 10 **k**-points. Elastic constants and deformation potential, required to compute the different scattering contributions, were computed using the same setup used for the geometry optimization and force constant calculations. Due to the strong dependence between the band gap and the high-frequency dielectric constant, our values have been corrected considering the linear correlation found between the computed and experimental values (see Supporting Information).

### Computational Cost

This study introduces a novel approach to reduce the computational resources needed for calculating thermal and electronic transport properties while still maintaining high accuracy. During the last few years, codes such as ElecTra,^26^ TransOpt,^40^ and AMSET^25^ have been developed, presenting a similar computational cost to other codes based on the constant relaxation approximation,^14^ but with an accuracy similar to the combination of Wannier functions and density functional perturbation theory, DFPT.^24^ For example, a comparison between AMSET and DFPT + Wannier reveals that the former has a significantly lower computational cost compared to that of the latter, with nearly 2 orders of magnitude difference.^25^ These codes have been combined with the solution of the BTE for phonons to compute the figure of merit of potential TE materials.^41−43^ However, the high computational cost linked to the calculation of high-order IFCs makes this task difficult to tackle for more than one or two materials.^44−46^ The use of a ML-based approach through the hiPhive package drastically reduces the number of calculations needed for extracting the high-order IFCs.^21,38^ For skutterudites, the number of single-point supercell calculations is reduced from more than 680 using the finite-difference approach to only 14 with hiPhive. Integrating AMSET and hiPhive within a high-throughput framework provides the opportunity to explore the large and complex chemical space of binary skutterudite in this work. We have estimated that during this work we have consumed around 2 × 10^5^ core hours using Intel(R) xeon(R) CPU E5-26700 @ 2.60 GHz; however, combining the finite-differences approach for κ_l_ and Wannier functions for electronic transport properties would have cost around 6 × 10^7^ core hours. This is a good example of how the combination of ML techniques and a high-throughput framework not only reduces the computational cost and automates the calculation of material properties but also democratizes research.^47−49^

## Results and Discussion

### Validation

#### Lattice Dynamics and Thermal Transport Properties

**Figure 1:**
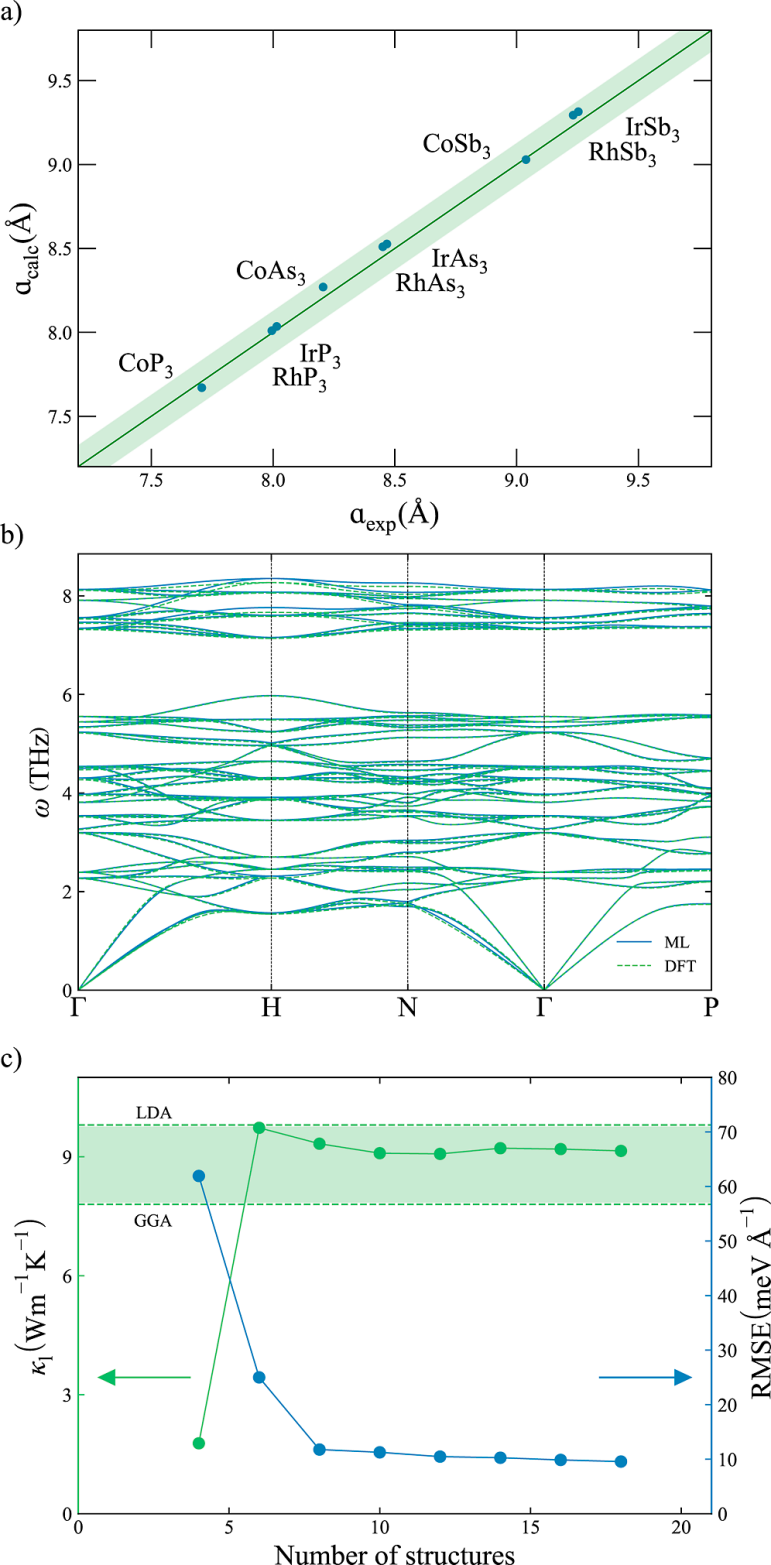
(a) Comparison of DFT-calculated vs experimental conventional cell parameters.^50−55^ The solid line represents perfect agreement, and the green shaded area has deviations of up to 2%. (b) Phonon dispersion curves for CoSb_3_ using DFT dashed (green line) and ML (blue line). (c) Calculated lattice thermal conductivity (green line) and RMSE (blue line) convergence with the number of distorted supercells during the training. Dashed green lines represent the values reported using LDA and PBE functionals using exclusively DFT calculations.^56^

A good description of interatomic distances represents one of the main requirements for obtaining accurate κ_l_ values. Experimental and calculated lattice parameters are compared in Figure 1a in order to evaluate the quality of the DFT methodology used for describing the structural properties of these materials. An excellent agreement is found between experimental and calculated lattice parameters, with errors below 2% in all cases.^50−55^ For instance, while LDA and PBE functionals tend to underestimate and overestimate, respectively, the experimental lattice parameter of CoSb_3_,^56^ PBE-D3 functional predicts a value much closer to the experimental value.

The exchange correlation functional is not the only variable to consider when optimizing the calculation of κ_l_ from first principles. Interaction cutoff radii are necessary to reduce the computational cost of the model. These cutoffs were defined based on coordination shells to guarantee transferability between materials that belong to the same structural prototype. Harmonic (second-order) force constant cutoffs are set to build the largest sphere that can be fit inside the supercell. Very good agreement was found between the phonon dispersion curves for CoSb_3_ obtained using exclusively DFT forces with VASP and phonopy and the results obtained by combining DFT calculations with ML to predict harmonic force constants (Figure 1b). Mean absolute error, MAE, and root-mean-square error, RMSE, are below 0.013 and 0.021 THz, respectively (see Supporting Information). The number of distorted supercells required to train the ML model also plays an important role, especially for third- and fourth-order FCs. We have found that 14 supercells are enough not only to obtain a RMSE in the forces below 15 meV/Å but also for converging the computed κ_l_ (Figure 1c). These results are in agreement with previous predictions of κ_l_ for CoSb_3_ using a more expensive computational approach, in which each force constant was extracted directly from DFT calculations by finite differences, resulting in a much larger number of distorted structures being required. A κ_l_ of 9.22 W/m K is obtained using the PBE-D3 functional, which is in between the results predicted with LDA and PBE functionals using similar cutoff values for third-order IFCs.^56^ This fact is consistent with the calculated lattice parameters for CoSb_3_, in which the PBE-D3 value is in between the results obtained with LDA and PBE functionals.^56^ Interaction cutoff distances below 6.5 Å tend to predict larger values of κ_l_.^58,59^ Guo et al. already demonstrated that harmonic IFCs almost vanish at a cutoff interatomic distance of 6.5 Å. The same trend was found using IFCs extracted from ML regression and obtaining converged values in the validation RMSE (see Supporting Information). All these calculated values are slightly below the experimental κ_l_ (10 W/m K)^57,60^ due to the softening of the vibrational modes at finite temperatures, a factor that is not included in the solution of the BTE.

#### Electronic Transport Properties

**Figure 2:**
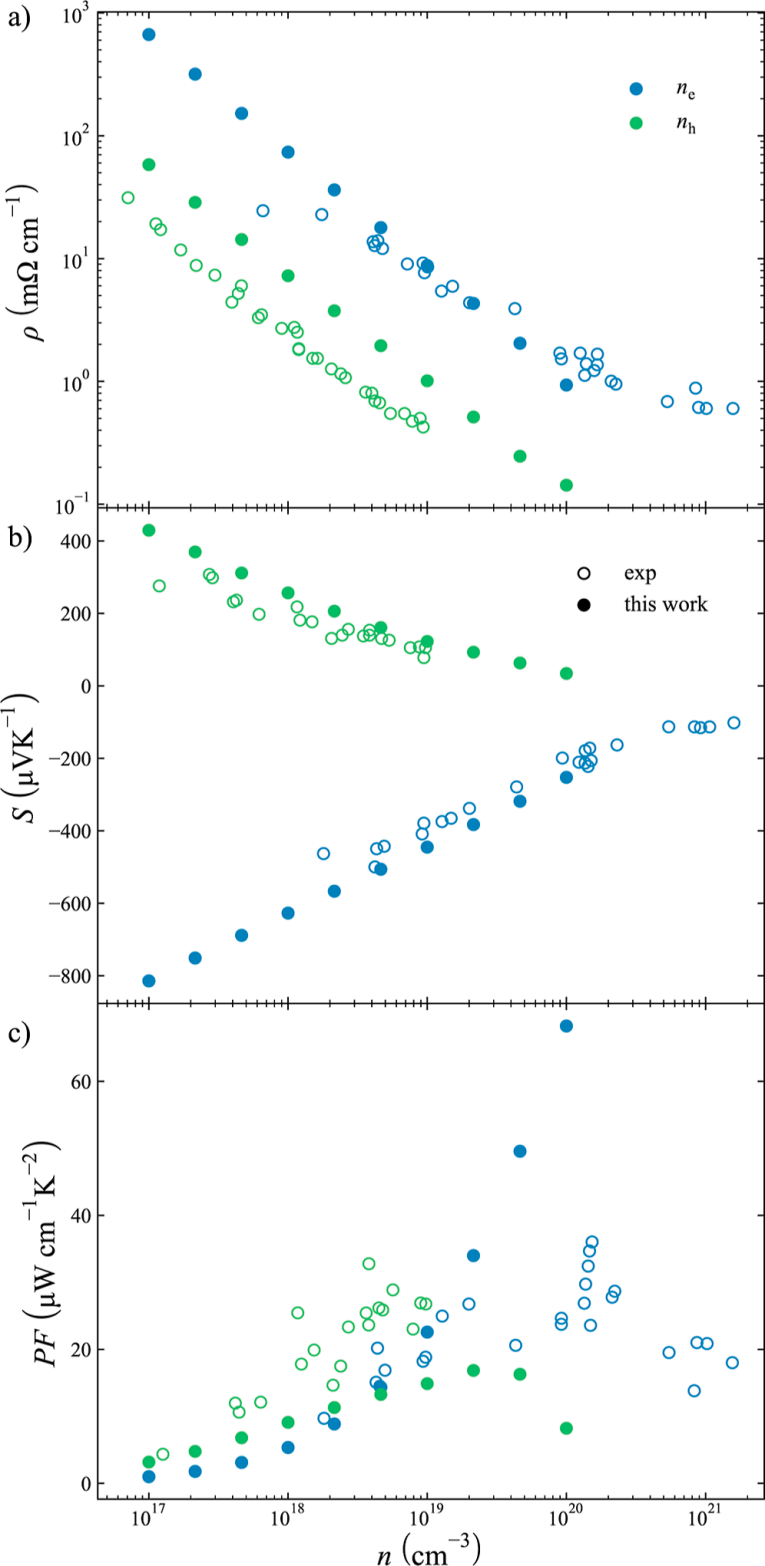
(a) Resistivity, ρ; (b) Seebeck coefficient, *S*; and (c) PF for CoSb_3_ at different carrier concentrations n at 300 K. Green and blue are used for p-type and n-type conduction, respectively. Calculated and reported experimental data^57^ are represented with solid and empty points, respectively.

Both p- and n-type skutterudites have been explored in a large range of carrier concentrations (10^17^–10^20^ cm^–3^) and temperatures (300–1000 K). Experimental electronic transport properties for single-crystal CoSb_3_ at 300 K have been used to evaluate the accuracy of this approach. While resistivity, ρ = 1/σ, is slightly overestimated for p-type CoSb_3_ in the whole range of concentrations, very good agreement is found for n-type CoSb_3_ with only small deviations at large carrier concentrations (Figure 2a). An overestimation of the calculated values of σ at high carrier concentrations is expected because the model does not explicitly include the electron scattering at point defects. Calculated *S* values also consistently match the experimental values^57^ at the whole range of concentrations (Figure 2b). The power factor, PF = *S*^2^σ, is slightly overestimated for p-type CoSb_3_, while the values for n-type CoSb_3_ are in good agreement with the experimental values, and deviations are only found at very high carrier concentrations (Figure 2c). Ionized scattering rates calculated in AMSET take into account the charge of the impurity and its concentration but do not include the effect of the impurity in the lattice and its potential modification in the band structure. That is why this work does not explore carrier concentrations above 10^20^ cm^–3^. Heavily doped systems require the creation of models where the impurity is explicitly included.^63^ This scenario cannot be implemented in a high-throughput framework because of (i) the large number of models (one specific model per dopant and carrier concentration) and (ii) the computational cost (very large cell units with low symmetry are required).

### Thermal Conductivity

**Figure 3:**
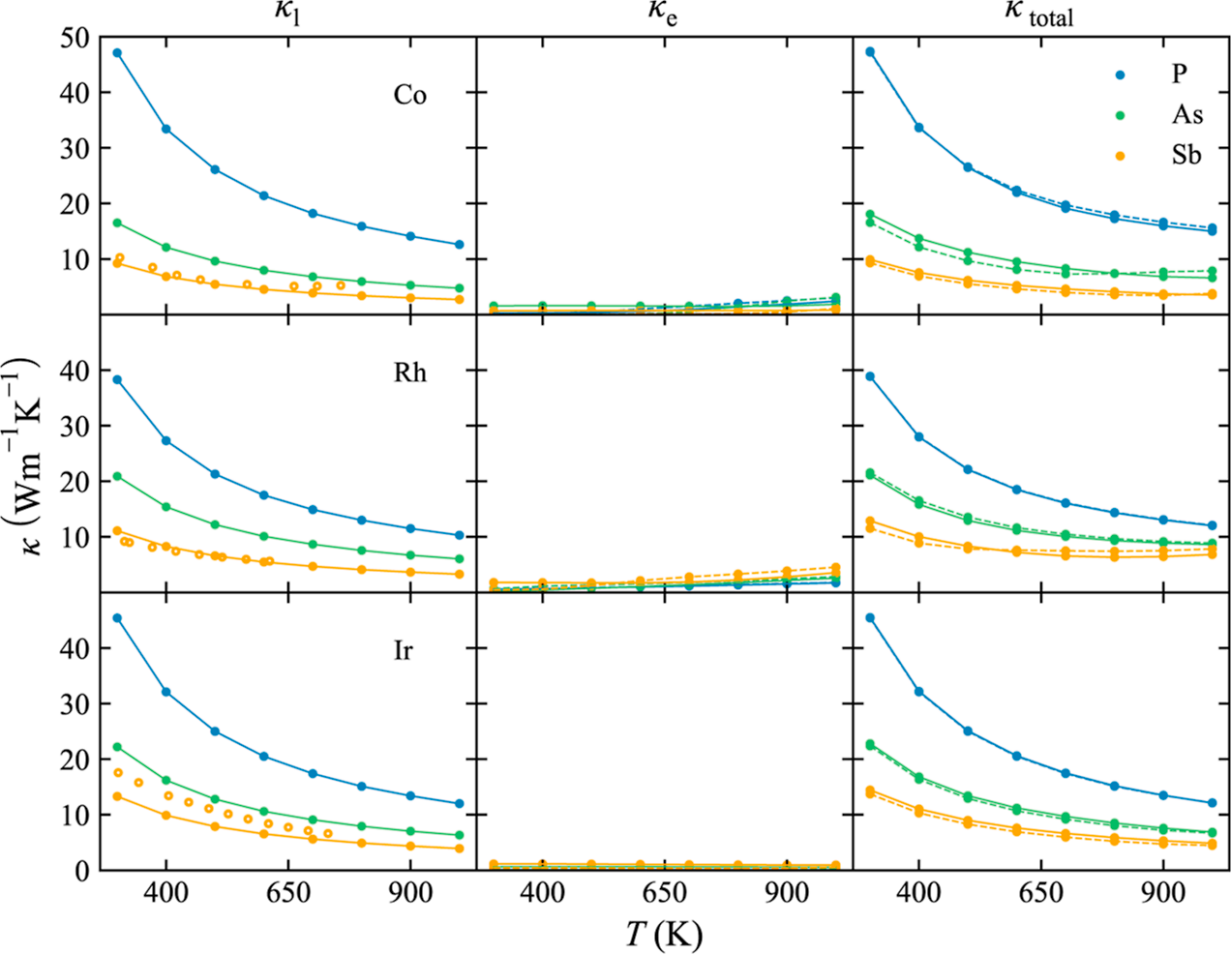
Calculated lattice (left column), electrical (central column), and total (right column) thermal conductivity of binary skutterudites. Phosphide, arsenide, and antimonide compounds are depicted in blue, green, and orange, respectively. Co, Rh, and Ir compounds are represented in the top, mid, and bottom rows, respectively. Experimental data are included using empty circles.^57,61,62^ The electronic contribution to the thermal conductivity, κ_e_, is depicted for the carrier concentration, *n* = 5 × 10^18^ cm^–3^. Solid lines represent p-type and dashed lines represent n-type skutterudites, respectively.

The computed thermal conductivity of MX_3_ (M = Co, Rh, Ir; X = P, As, Sb) skutterudites is depicted in Figure 3. Being semiconductors, as most reported skutterudites, the studied materials present very low κ_e_ for both p-type (solid lines) and n-type (dashed lines), so κ_total_ (=κ_l_ + κ_e_) is governed by κ_l_ at low and medium temperatures. This opens the door to the optimization of *ZT* through the reduction of κ_l_. The contribution of κ_e_ on κ_total_ is only significant at very high temperatures, where κ_l_ is drastically reduced. For instance, κ_e_ represents around 50% of κ_total_ at 1000 K for RhSb_3_.

**Figure 4:**
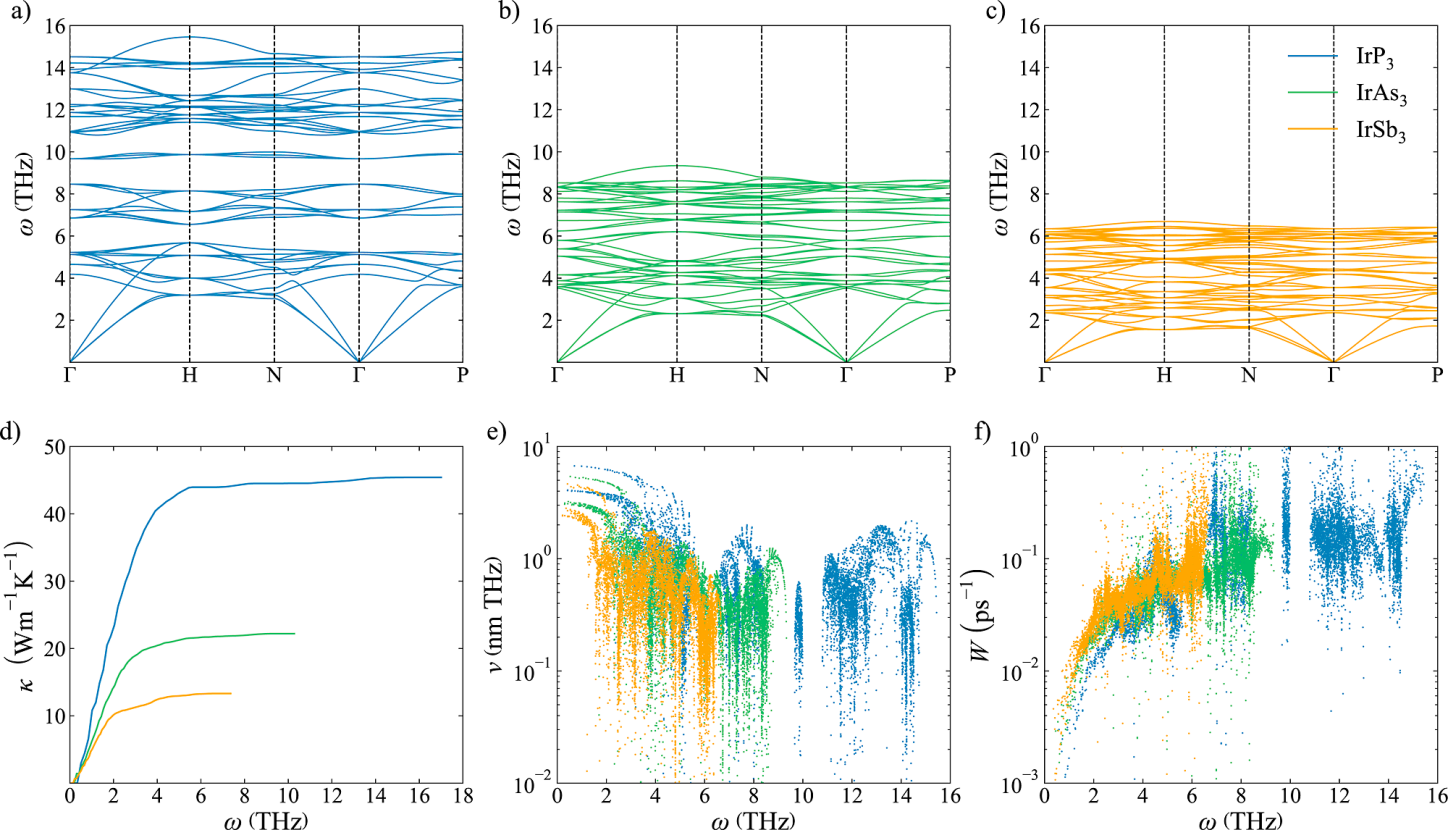
(a–c) Phonon dispersion curves for IrP_3_, IrAs_3_, and IrSb_3_, respectively. (d) Cumulative κ_l_; (e) group velocities, *v*; and (f) scattering rates, *W*, for IrP_3_ (blue), IrAs_3_ (green), and IrSb_3_ (orange).

Quantitatively, our predictions for κ_l_ are in good agreement with the experimental data in most of the temperature range when available.^57,61,62^ The largest deviations are found for IrSb_3_ at low temperatures. Mingo et al. obtained lattice thermal conductivity values closer to the experimental values using the BTE combined with LDA calculations and a shorter cutoff distance.^59^ Although LDA predicts the lattice parameter closer to the experimental parameter than PBE-VdW, this functional should be used carefully with low-band gap semiconductors, as is the case for skutterudites. Relativistic effects attached to heavy elements such as Ir can also modify IFCs. Wu et al. demonstrated that spin–orbit interactions have a significant impact on the calculation of the thermal transport properties of SnSe, increasing κ_l_.^64^ Considering the high computational cost of including spin–orbit coupling in the supercell calculations and the fact that the largest deviations in IrSb_3_ are located at low temperatures where *ZT* is presumably low, we have opted for not including relativistic effects. However, it is even more important to understand the wide range of values obtained for κ_l_. While there are very few changes in κ_e_ based on chemical compositions, κ_l_ seems to be strongly modified by both the cation and the anion involved. The role of the pnictogenide follows the same trend for all of the cations: κ_l_(MP_3_) > κ_l_(MAs_3_) > κ_l_(MSb_3_). This trend is explained by the reduction in the frequencies of the optical modes when P is substituted by As or Sb (Figure 4a–c). Phonon dispersion curves show how the largest frequency is reduced from 15 THz for IrP_3_ to approximately 6.3 THz for IrSb_3_. This results in a large increase in the phonon density of states (DOS) because of the overlap between the bands of different vibrational modes. The avoided crossing between the low-lying optical modes and the acoustic modes that are the main contributors to κ_l_ (Figure 4d) produces a flattening of the acoustic modes, reducing their group velocities (Figure 4e) and their contribution to κ_l_. Additionally, the higher phonon DOS at low frequencies facilitates the energy conservation required for phonon–phonon scattering processes. A larger number of scattering processes in IrAs_3_ and IrSb_3_ with respect to IrP_3_ result in a growth of the scattering rates (Figure 4f) and a reduction of κ_l_.

**Figure 5:**
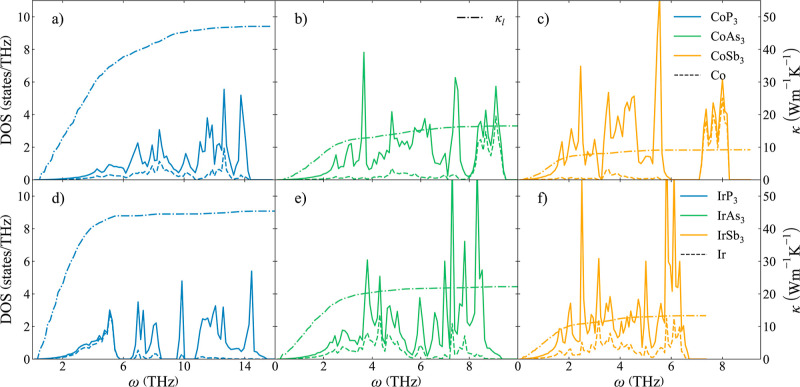
Phonon DOS (solid lines) and cumulative lattice thermal conductivity, κ_l_, (dotted–dashed lines) for Co- and Ir-based binary skutterudites. Projected DOS on the cations is depicted with dashed lines.

To understand the role of the cation in the lattice thermal conductivity, it is necessary to examine the atoms involved in the acoustic modes (Figure 5). For P-based skutterudites, cations present larger masses than P, so Co, Rh, and Ir are the main contributors to the acoustic band (Figure 5a–d). Thus, lattice thermal conductivity is dominated by the cation atomic mass, so κ_l_(CoP_3_) > κ_l_(RhP_3_) > κ_l_(IrP_3_). However, for Sb-based skutterudites, Sb presents the largest participation in the acoustic modes in all three compounds (Figure 5c–f). That is why the difference between the κ_l_ of CoSb_3_, RhSb_3_, and IrSb_3_ is considerably lower compared to that of their P-based counterparts. The atomic mass difference between the cation and the anion plays a secondary role and explains that the trend is reverted for Sb-skutterudites, resulting in κ_l_(IrSb_3_) > κ_l_(RhSb_3_) > κ_l_(CoSb_3_).

The role of each phonon scattering mechanism also needs to be clarified. Grain boundary effects are analyzed at the end of the section, so here we will focus on the scattering processes of single crystals. Three phonon scattering and inelastic (isotopic) scattering processes are both included in the calculation of κ_l_. Some of the compounds explored here do not include inelastic scattering because Co, Rh, P, and As only present one natural isotope. That is why the lattice thermal conductivity of isotopically pure IrSb_3_ has been computed. When inelastic scattering is removed from the BTE solution, κ_l_ only increases around 1% (0.15 W/m K) for IrSb_3_ at 300 K, which demonstrates that three-phonon scattering is the major mechanism that governs thermal transport in single-crystal skutterudites.

### Power Factor and Carrier Mobility

**Figure 6:**
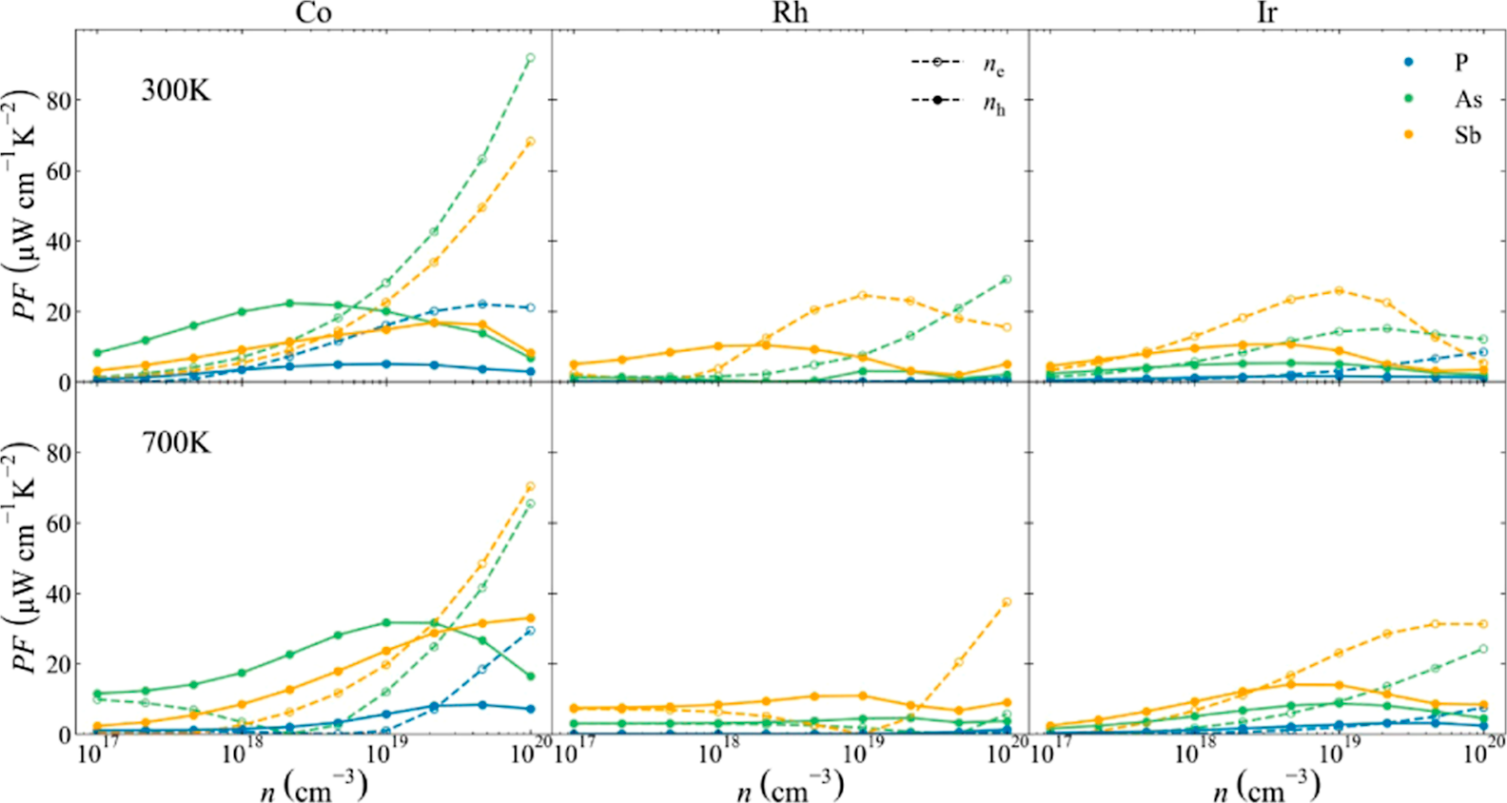
PF of Co- (left), Rh- (middle), and Ir-based (right) skutterudites at 300 K (top row) and 700 K (bottom row). Phosphide, arsenide, and antimonide compounds are depicted in blue, green, and orange, respectively. p- and n-type semiconductors are depicted using solid and dashed lines, respectively.

Due to the strong interdependence of *S* and σ, we will analyze both properties together, examining the PF (Figure 6). Different trends can be extracted from Figure 6 based on the behavior of PF with respect to temperature, carrier concentration, and composition. For instance, PF seems to be higher at 300 K than at 700 K at moderate carrier concentrations. However, the two key variables that drastically change PF are composition and type of carrier: n-type semiconductors present a larger PF than their p-type counterparts. These trends will be analyzed and explained in more detail in the next section. Regarding composition, there is a clear trend with Co- and Sb-based skutterudites presenting the largest PF values, particularly both n-type CoSb_3_ and CoAs_3_ compounds. In CoSb_3_, this behavior has been previously explained by the quasi-degeneracy between the Co–Sb and Sb–Sb electronic levels at the conduction band, which favors band convergence in n-type skutterudites.^65^ In order to confirm this trend, the HSE band structures for the three compounds with the largest PF (CoSb_3_, CoAs_3_, and IrSb_3_) have been computed (see Supporting Information). All valence bands present a single carrier pocket centered at Γ, while the conduction band presents different carrier pockets. CoAs_3_ and CoSb_3_ present a secondary pocket between Γ and *N*. This secondary pocket is not well-defined for IrSb_3_, which reduces its PF with respect to those of the other two compounds at high carrier concentrations. Another carrier pocket can be found close to the conduction band edge at *H*. This minimum is not usually properly characterized using GGA or meta-GGA functionals but has already been described when hybrid functionals are used.^66^

Once the main trends are analyzed based on the chemical composition and carrier concentration, it is crucial to examine the role of each type of scattering mechanism included in the theoretical model. This step is the key to rationalizing how electronic transport properties can be modified in skutterudites. An effective approach for this task is to evaluate the contributions of each scattering mechanism to the carrier mobility, μ. It looks like polar optical phonon scattering is the mechanism that dominates carrier mobility in skutterudites over the whole studied range of temperatures and carrier concentrations (see Supporting Information). Due to the polarization induced by the optical vibration modes, the electrons are scattered through the interaction of the Coulomb field of the lattice polarization waves. Only at very high carrier concentrations do ionized impurity scattering processes contribute to total mobility. This fact again highlights the strong correlation between the phonon vibrational structure and transport properties. While acoustic modes are the key to thermal transport properties, optic modes also play an important role in determining the electron mobility of the skutterudite.

### TE Figure of Merit

**Figure 7:**
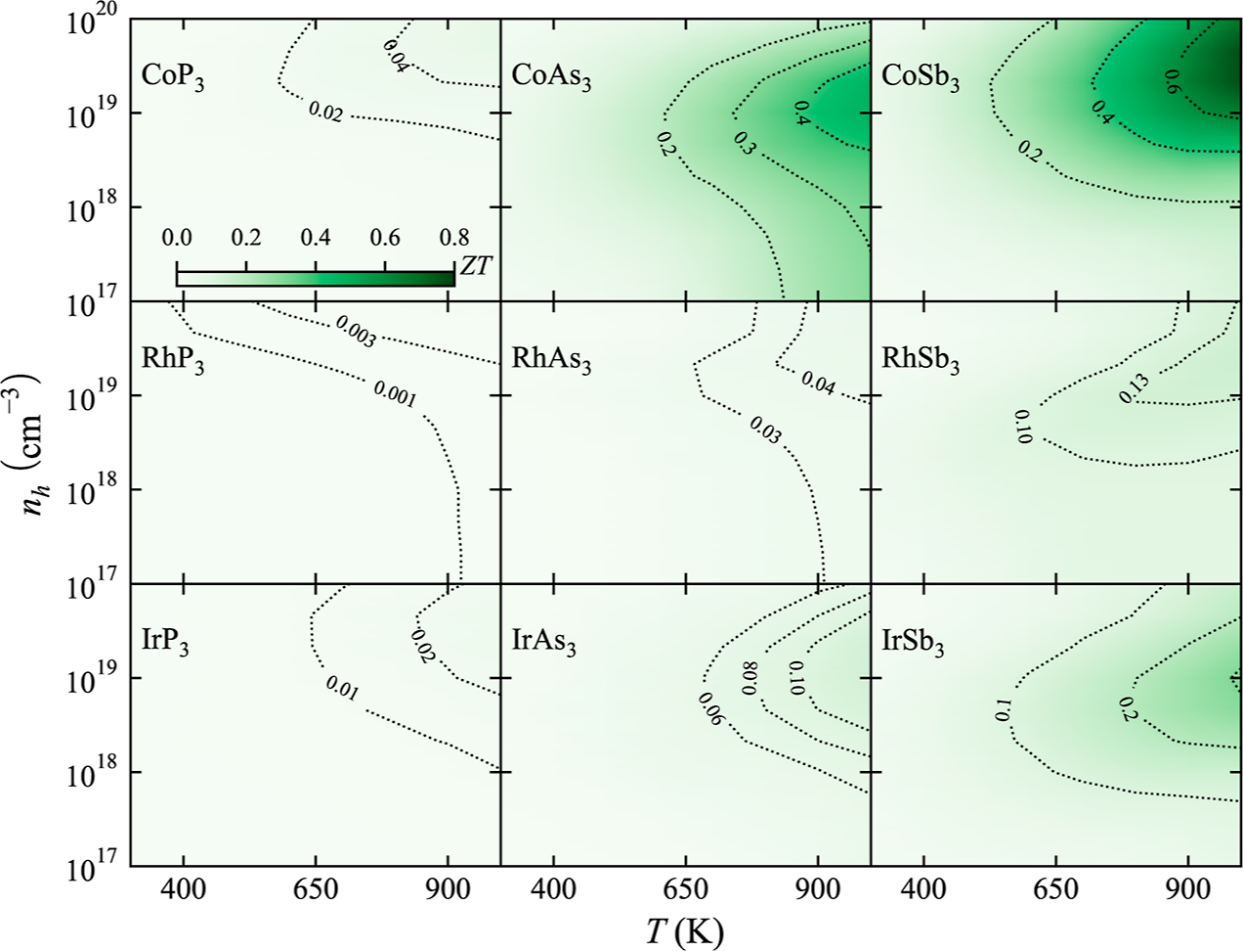
TE figure of merit, *ZT*, dependence on temperature, *T*, and carrier concentration, *n*_h_, for p-type binary skutterudites.

**Figure 8:**
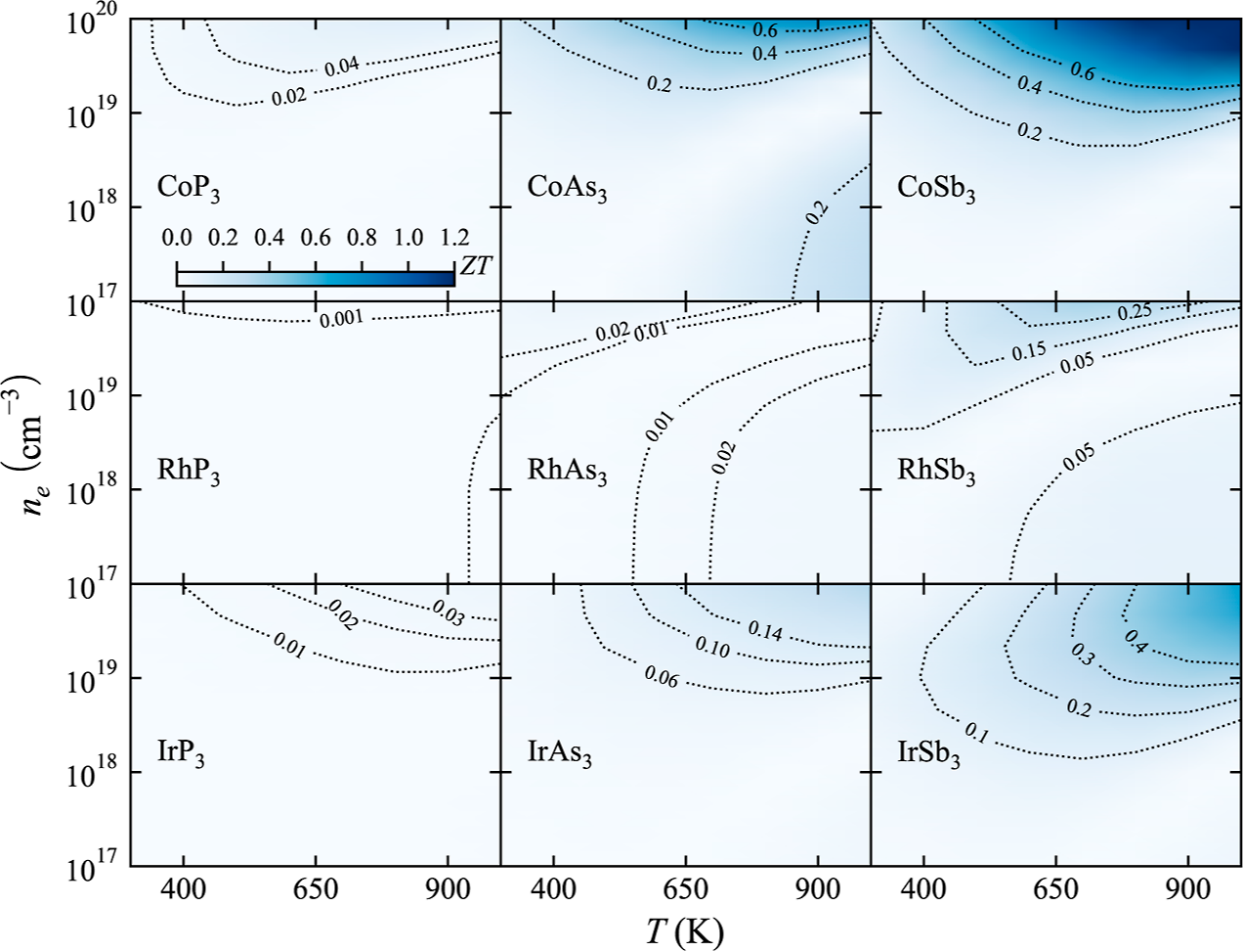
TE figure of merit, *ZT*, dependence on temperature, *T*, and carrier concentration, *n*_e_, for n-type binary skutterudites.

The TE figure of merit, *ZT*, of p-type (Figure 7) and n-type (Figure 8) binary skutterudites has been calculated by combining κ and PF in a wide range of temperatures and carrier concentrations, *n*. For both types of semiconductors, it was found that only three present *ZT* > 0.25 (CoAs_3_, CoSb_3_, and IrSb_3_). The low *ZT* values were expected due to the lack of a rattler that would reduce κ_l_ in filled skutterudites. Our predictions are in quantitative agreement with experimental results reported for CoSb_3_.^67−69^

It is well established that n-type CoSb_3_ usually presents a higher *ZT* than its p-type counterpart.^70^ For instance, previous works have obtained *ZT* values between 0.2 and 0.05 for p-type CoSb_3_ at 600 K depending on the carrier concentration.^67−69^ Calculated *ZT* values in this work are found in the same range when *n* < 10^19^ cm^–3^. *ZT* values for n-type CoSb_3_ are much larger at 600 K^71^ (0.43 when *n* = 2 × 10^19^ cm^–3^), in good agreement with experimentally reported values (0.45–0.6).^71^

In addition to the quantitative difference in *ZT* between n-type and p-type skutterudites, important differences were found in their behavior with the temperature and carrier concentration. *ZT* values for p-type skutterudites do not change drastically based on the carrier concentration, and it seems that temperature plays a critical role in maximizing *ZT*. Large values of *ZT* are obtained only at high carrier concentrations for p-type skutterudites. This different behavior observed in the contour plots in Figures 7 and 8 necessarily stems from the different topologies of the valence and conduction bands.

It is well-known that the favorable electronic band structure in n-type CoSb_3_ cannot be only attributed to the 3-fold degenerated states at the bottom of the conduction band.^72^ There is a secondary conducting carrier pocket that is responsible for the excellent TE performance of n-type CoSb_3_. However, high carrier concentrations are needed to populate this secondary pocket, which explains why *ZT* only gets larger values when *n* > 1 × 10^19^ cm^–3^. That is why n-type skutterudite performance is driven by its electronic transport properties. There are not important secondary bands in the valence band, so modifying the carrier concentration does not affect *ZT* too much, and the reduction of κ_l_ when temperature is increased is the term that governs the optimization of the TE performance.

**Figure 9:**
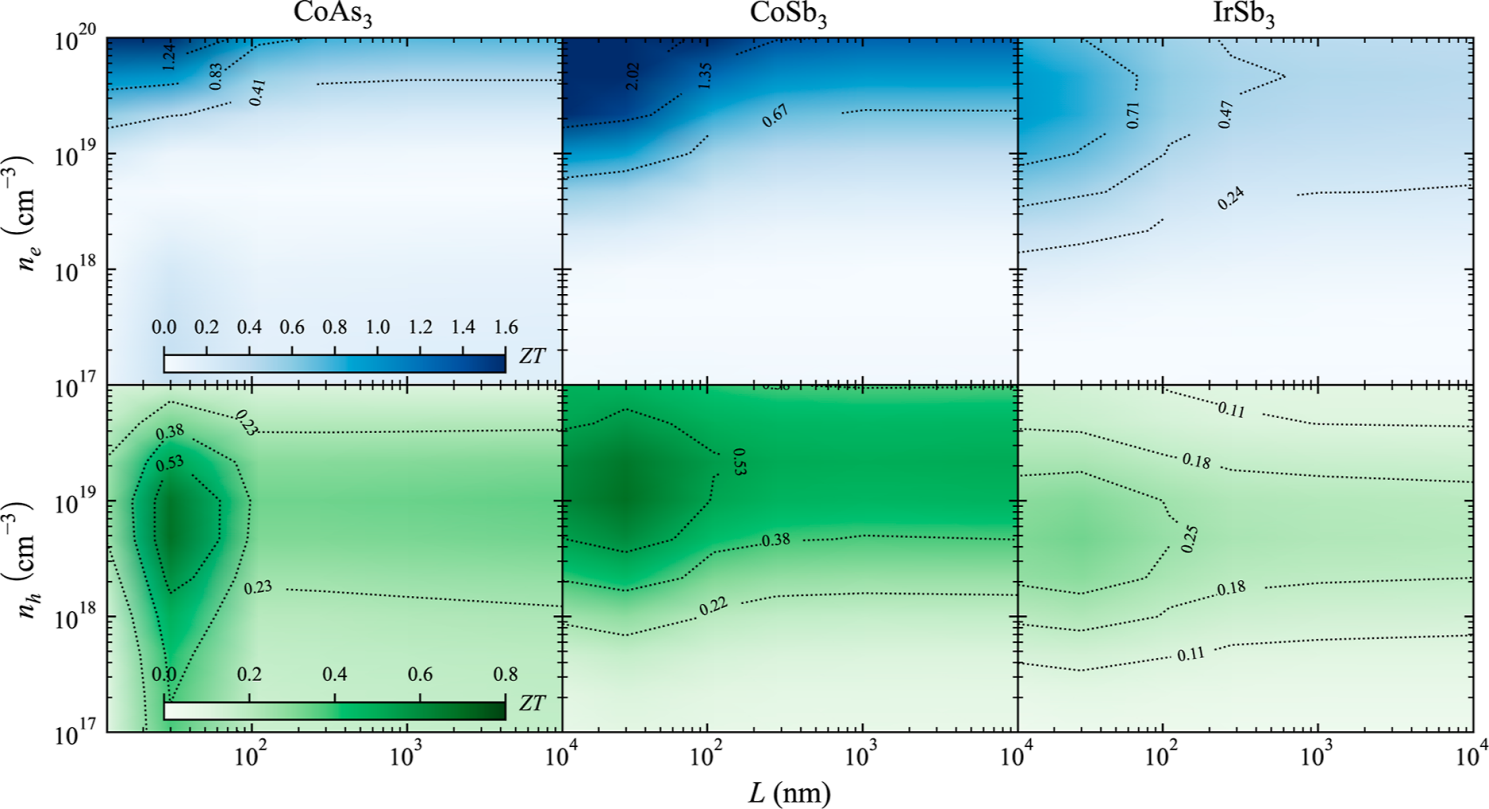
TE figure of merit, *ZT*, dependence on grain size, *L*, and carrier concentration, *n*, for n-type (blue) and p-type (green) selected binary skutterudites at 800 K.

Optimizing *ZT* involves considering variables beyond just the temperature and carrier concentration. The microstructure plays a crucial role in determining the thermal and electronic transport properties of skutterudites. Several methods for synthesizing and processing skutterudites have been reported, leading to significant enhancements in their TE performance.^73,74^ Techniques such as spark plasma sintering^75^ or cold sintering process^76^ can tailor the skutterudite microstructure and improve *ZT*. One of the techniques that has yielded significant improvements is high-pressure torsion, HPT,^77^ which introduces many grain boundaries as well as dislocations in the structure, enhancing *ZT* values up to a factor of 2 for some skutterudites.^78^ The methodology used in this study can also take into account the influence of grain boundaries on the electronic and thermal transport properties of materials.^79^ We opted to study the three binary skutterudites that exhibit the highest *ZT* values at 800 K: CoAs_3_, IrSb_3_, and CoSb_3_ (Figure 9). Some differences can be observed in the *ZT* contour plots of these three skutterudites. It is worth noting that the alignment of contour lines parallel to the *x*-axis suggests that reducing the grain size, *L*, does not have a significant effect on *ZT*. While n-type IrSb_3_*ZT* values are improved below 600–500 nm, n-type CoAs_3_ and CoSb_3_ show little change in *ZT* with grain size reduction until grain sizes are below 100 nm. This trend is similar for p-type semiconductors where IrSb_3_*ZT* values are modified for grain sizes around 300 nm and below, while CoAs_3_ and CoSb_3_ show little change until grain sizes are below 100 nm. These results align well with CoSb_3_-based samples processed through HPT where a drastic improvement of *ZT* was achieved with a grain size of around 50 nm.^78^ In all cases, the improvement of *ZT* is attributed to a stronger reduction of the lattice thermal conductivity compared to that of the electrical conductivity. Grain boundaries are known to scatter phonons and electrons, effectively reducing thermal and electrical conductivity. However, if phonon mean free paths are considerably longer than electron mean free paths, then it is possible to identify a specific range of grain sizes where *ZT* can be increased. For instance, p-type CoSb_3_ thermal conductivity is drastically reduced in grain sizes below 100 nm; however, electrical conductivity is not noticeably reduced until the grain sizes are below 30 nm at 800 K. The case of n-type IrSb_3_ is particularly interesting as larger grain sizes have the potential to significantly increase *ZT* values. To the best of our knowledge, this phenomenon has not been previously reported and is linked to the longer phonon mean free path of IrSb_3_ compared to that of other binary skutterudites.

## Conclusions

The critical role of material transport properties in different technological applications has led to the pressing need to develop new strategies to predict them accurately and systematically. For instance, efficient TE materials require the simultaneous optimization of their electronic and thermal transport properties. In this work, we present a new high-throughput framework that combines state-of-the-art ab initio methodologies with ML to predict σ, *S*, κ, and *ZT*, minimizing the computational cost. To demonstrate the robustness of this approach in exploring large physical and chemical spaces, the transport properties and *ZT* of binary skutterudites have been calculated. Although most high-performance skutterudites present fillers, TE optimization is not exclusively related to the exploration of chemical spaces. Transport properties are not only strongly dependent on composition but also on other physical variables such as temperature, carrier concentration, or nanostructure. The calculated lattice thermal conductivity is in good agreement with experimental reports when available. Phonon dispersion curves, phonon DOS, group velocities, and scattering rates are combined to rationalize the predicted values. The presence of heavy anions, such as Sb, plays the primary role in reducing κ_l_ through a contraction of the group velocities and an enhancement of the number of scattering processes. The mass difference between Sb and the cation plays a secondary role that explains why CoSb_3_ presents the lowest κ_l_ among all of the explored materials. The PF is especially high for n-type semiconductors where Sb is the anion due to the band convergence that enhances the Seebeck coefficient. *ZT* can be optimized through an exploration of *T*, *n*, and *L* when electronic and thermal transport properties are combined. For n-type semiconductors, the carrier concentration is the driving force for *ZT* maximization because PF is strongly enlarged when the secondary conduction band is populated. On the contrary, p-type skutterudites are optimized at high temperatures, where κ_l_ is reduced. It is demonstrated that nanostructuring is a good strategy to improve *ZT* in skutterudites. Our results show the same trend as experimental reports in which *ZT* is drastically enhanced with grain sizes below 50 nm and identify n-type IrSb_3_ as a good candidate to enlarge its *ZT* by nanostructuring. This work demonstrates the importance of the development of new strategies to accurately and systematically predict transport properties, connecting them with synthetic and operando variables. These new tools not only open the door to exploring large chemical spaces but also extract design principles based on the chemical and physical phenomena that govern these properties.

## Data Availability

Data are available at the ZENODO repository (doi:10.5281/zenodo.8042323).

## Supporting Information Available

IFC validation, nonanalytical contribution effects on KL, electronic transport properties—AMSET, cutoff convergence, electronic band structures, and carrier mobility decomposition in the different scattering mechanisms (PDF)
