# Supplementary material for: High-Throughput Prediction of the Thermal and Electronic Transport Properties of Large Physical and Chemical Spaces Accelerated by Machine Learning: Charting the ZT of Binary Skutterudites
Source: ACS Appl Mater Interfaces. 2024 Jan 22;16(4):4606–17. doi: 10.1021/acsami.3c15741 (PMC10835667; doi:10.1021/acsami.3c15741)
Supplement: Supplementary file 1 — am3c15741_si_001.pdf [file am3c15741_si_001.pdf]

Supplementary Material: High-throughput Prediction of the Thermal and Electronic Transport Properties of Large Physical and Chemical Spaces Accelerated by Machine Learning: Charting the ZT of Binary Skutterudites

Julia Santana-Andreo,[†,§] Antonio M. Márquez,[†] Jose J. Plata,[†] Ernesto J. Blancas,[†] José-Luis González-Sánchez,[‡] Javier Fdez. Sanz,[†] and Pinku Nath[*,¶]

†*Departamento de Química Física, Facultad de Química, Universidad de Sevilla, 41012 Seville, Spain*

‡*Department of Computer Systems Engineering and Telematics, University of Extremadura, School of Technology, 10003 Cáceres, Extremadura, Spain*

¶*Institute for Chemical Reaction Design and Discovery (WPI-ICReDD), Hokkaido University, 060-0808 Sapporo, Japan*

§*Current address: Institute of Physics, Carl-von-Ossietzky, Universität Oldenburg, 26129 Oldenburg, Germany*

E-mail: pnath@icredd.hokudai.ac.jp

Abstract

Thermal and electronic transport properties are the key to many technological

applications of materials. Thermoelectric, TE, materials can be considered a singular case in which not only one but three different transport properties are combined to describe their performance through their TE figure of merit, ZT. Despite the availability of high-throughput experimental techniques, synthesizing, characterizing, and measuring properties of samples with numerous variables affecting ZT is not a cost- or time-efficient approach to lead this strategy. The significance of Computational Materials Science in discovering new TE materials has run in parallel to the development of new frameworks and methodologies to compute the electron and thermal transport properties linked to ZT. Nevertheless, the trade-off between computational cost and accuracy has hindered the reliable prediction of TE performance for large chemical spaces. In this work, we present for first the time the combination of new ab-initio methodologies to predict transport properties with machine learning and a high-throughput framework to establish a solid foundation for the accurate prediction of thermal and electron transport properties. This strategy is applied to a whole family of materials, binary skutterudites, which are well-known as good TE candidates. Following this methodology, it is not only possible to connect ZT with the experimental synthetic (carrier concentration and grain size) and operando (temperature) variables but also to understand the physical and chemical phenomena that act as driving forces in the maximization of ZT for p-type and n-type binary skutterudites.

IFCs validation

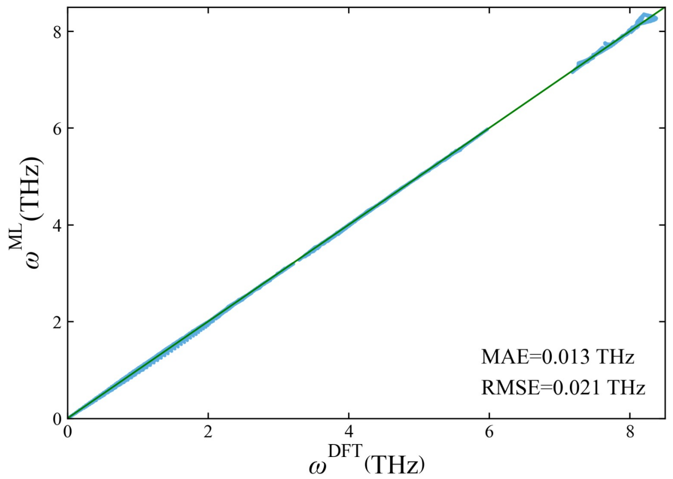

Figure S1: Comparison between DFT and ML frequencies for $CoSb_3$.

Non-analytical contributions

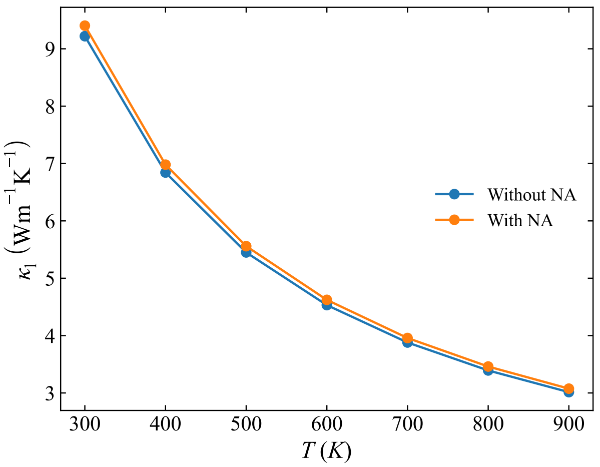

Figure S2: Lattice thermal conductivity for $CoSb_3$ with (orange) and without (blue) non-analytical contributions.

Electronic transport properties - AMSET

AMSET code solves the BTE for electrons without the RTA. Scattering rates are calculated using the Matthiesen's rule:

$$\frac{1}{\tau_e} = \frac{1}{\tau^{\mathrm{ADP}}} + \frac{1}{\tau^{\mathrm{IMP}}} + \frac{1}{\tau^{\mathrm{POP}}} + \frac{1}{\tau^{\mathrm{MFP}}}, \tag{1}$$

where τ^{ADP}, τ^{IMP}, τ^{ADP} and τ^{MFP} represent the scattering times due to the acoustic deformation potential, ionized impurities, polar optical phonons and grain boundaries, respectively. Piezoelectric scattering has not been included due to the centrosymmetric nature of skutterudites. The mode-dependent scattering rates, from state $|n\mathbf{k}\rangle$ to state $|m\mathbf{k}+\mathbf{q}\rangle$, are calculated using the Fermi's golden rule:

$$\tilde{\tau}^{-1}_{n\mathbf{k}\to m\mathbf{k}+\mathbf{q}} = \frac{2\pi}{\hbar} \left|g_{nm}(\mathbf{k},\mathbf{q})\right|^2 \delta\left(\varepsilon_{n\mathbf{k}} - \varepsilon_{m\mathbf{k}+\mathbf{q}}\right) \tag{2}$$

with ε being the electron energy, δ the Dirac delta function, and g the coupling matrix element. Electron transport properties were computed by the generalized transport coefficients,

$$L^n_{\alpha\beta} = e^2 \int \sum_{\alpha\beta}(\varepsilon)\left(\varepsilon - \varepsilon_F\right)^n \left[-\frac{\partial f^0}{\partial \varepsilon}\right] d\varepsilon, \tag{3}$$

where α and β represent cartesian coordinates, $\sum_{\alpha\beta}(\varepsilon)$ is the spectral conductivity, ε_F is the Fermi level at a doping concentration and temperature and f^0 is the Fermi-Dirac distribution. Finally, electronic transport properties are calculated as,

$$\sigma_{\alpha\beta} = L^0_{\alpha\beta}, \tag{4}$$

$$S_{\alpha\beta} = \frac{1}{eT}\frac{L^1_{\alpha\beta}}{L^0_{\alpha\beta}}, \tag{5}$$

$$\kappa_{\alpha\beta} = \frac{1}{e^2T}\left[\frac{\left(L^1_{\alpha\beta}\right)^2}{L^0_{\alpha\beta}} - L^2_{\alpha\beta}\right]. \tag{6}$$

Required material parameters for the calculation of scattering times are obtained through DFT calculations. Dense uniform band structure and wave function coefficients are obtained through single point calculations of the fully relaxed primitive cells. Wavefunction was considered converged when the energy difference between two consecutive electronic steps was smaller than 10^{-8} eV, using a dense mesh of $10 \times 10 \times 10$ **k**-points and the HSE06 functional proposed by Heyd *et al.*[1] Deformation potential, $\mathbf{D}_{n\mathbf{k}}$, is calculated as,

$$\mathbf{D}_{n\mathbf{k}} = \delta\varepsilon_{n\mathbf{k}}/\delta S_{\alpha\beta}, \tag{7}$$

where $\mathbf{S}$ is the uniform stress tensor and $\varepsilon_{n\mathbf{k}}$ is the energy of a band in a specific **k**-point. The deformation potential is averaged over contraction (–0.5 %) and expansion (+0.5 %) of the lattice and calculated separately for each component of the strain tensor. Eigenvalues are aligned to the average energy level of the core states which are calculated using the initial-state approximation.[2,3] Calculated dielectric constants and effective polar phonon frequency are obtained using density functional perturbation theory (DFPT)[4,5] using the PBE functional.[6] Due to the strong dependence between the band gap and the high-frequency dielectric constant, our values have been corrected considering the linear correlation found between the computed and experimental values (Fig. 3). Effective phonon frequency is determined from the phonon frequencies and phonon eigenvectors. To capture scattering from the full phonon band structure in a single phonon frequency, each phonon mode is weighted by the dipole moment.

Transport properties calculations were conducted under *p*- and *n*-type doping conditions within a range from 10^{17} to 10^{20} cm^{-3}, and the temperature range from 300 to 1000 K. Ionized impurity scattering was calculated using a charge of ± 1 for the impurity. In order to ensure converged properties, interpolation factor was set to 50 for all calculations and the energy cutoff used to determine which bands to include in the interpolation and scattering rate calculation was set to 1.5 eV.

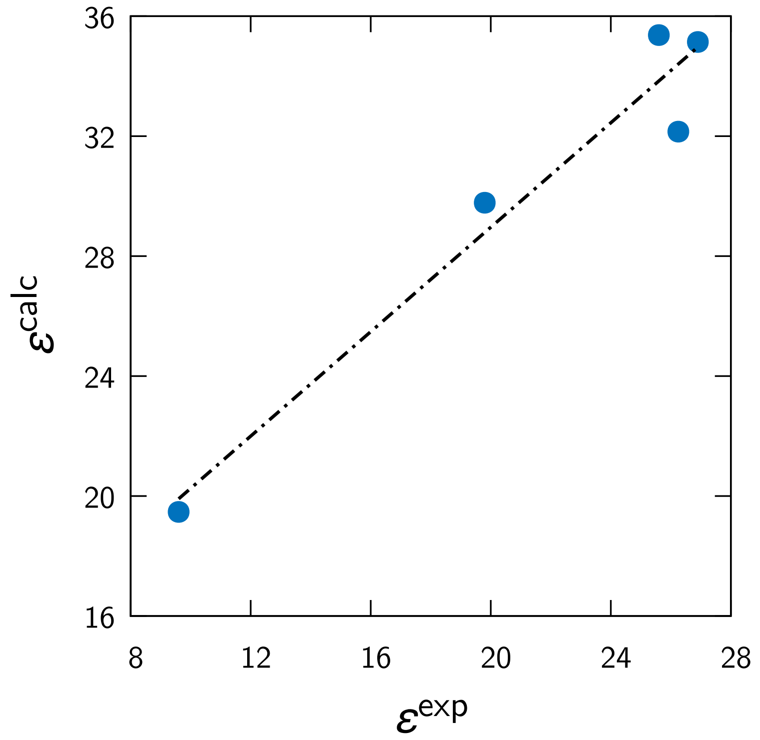

Figure S3: Comparison and correlation between experimental and calculated dielectric constants.

Cutoff

Band structure

Carrier mobility

References

(1) Heyd, J.; Scuseria, G. E.; Ernzerhof, M. Erratum: "Hybrid Functionals Based on a Screened Coulomb Potential" [J. Chem. Phys.118, 8207 (2003)]. *J. Chem. Phys.* **2006**, *124*.

(2) Lizzit, S.; Baraldi, A.; Groso, A.; Reuter, K.; Ganduglia-Pirovano, M. V.; Stampfl, C.;

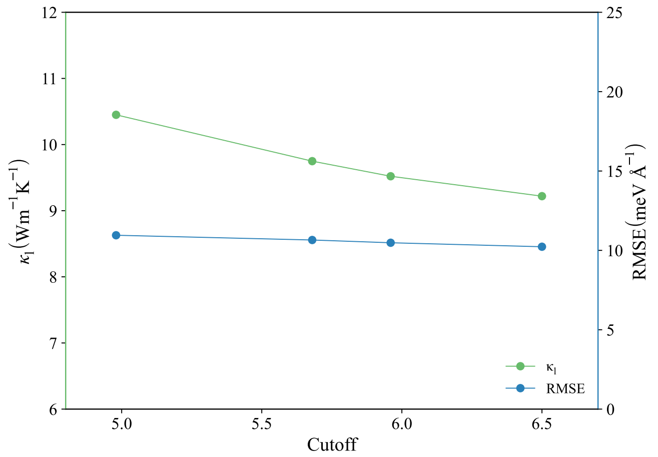

Figure S4: Calculated lattice thermal conductivity (green line) and RMSE (blue line) convergence with interaction cutoff distance for IFCs.

Scheffler, M.; Stichler, M.; Keller, C.; Wurth, W.; Menzel, D. Surface core-level shifts of clean and oxygen-covered Ru(0001). *Phys. Rev. B* **2001**, *63*, 205419.

(3) Köhler, L.; Kresse, G. Density functional study of CO on Rh(111). *Phys. Rev. B* **2004**, *70*, 165405.

(4) Baroni, S.; Resta, R. Ab initio calculation of the macroscopic dielectric constant in silicon. *Phys. Rev. B* **1986**, *33*, 7017–7021.

(5) Gajdoš, M.; Hummer, K.; Kresse, G.; Furthmüller, J.; Bechstedt, F. Linear Optical Properties in the Projector-Augmented Wave Methodology. *Phys. Rev. B* **2006**, *73*, 045112.

(6) Hammer, B.; Hansen, L. B.; Nørskov, J. K. Improved Adsorption Energetics Within Density-Functional Theory Using Revised Perdew-Burke-Ernzerhof Functionals. *Phys. Rev. B* **1999**, *59*, 7413–7421.

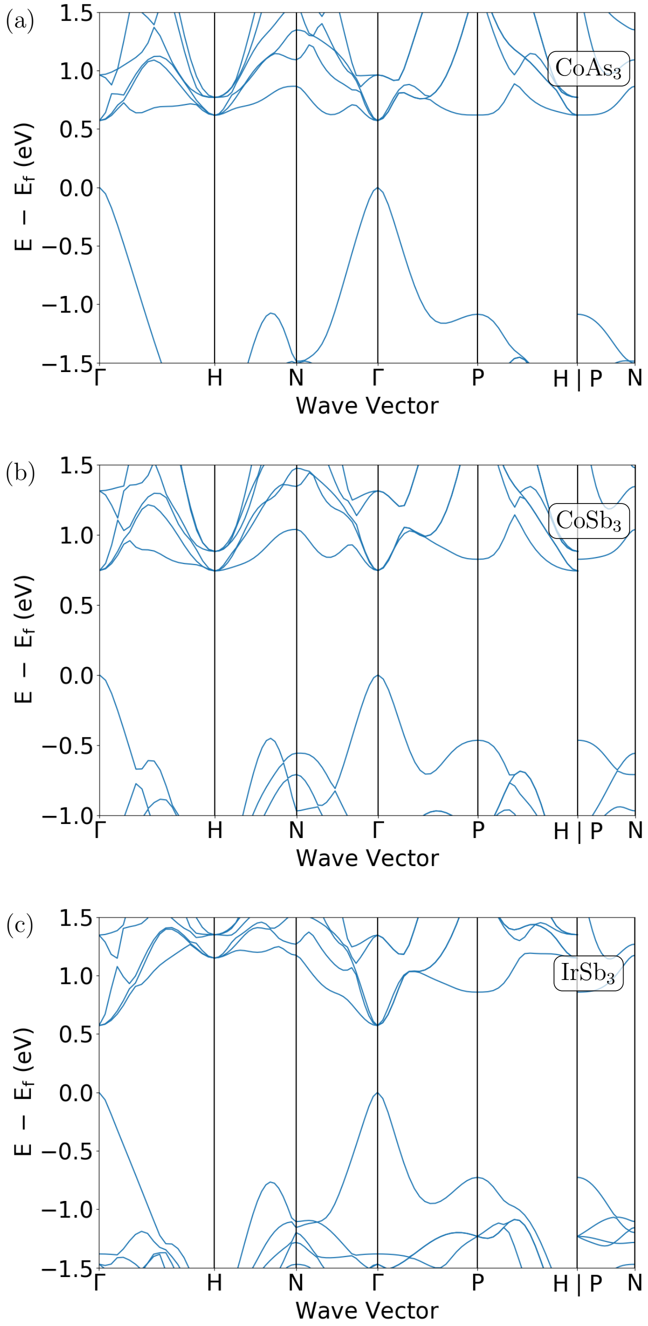

Figure S5: Electronic HSE band structure for $CoAs_3$, $CoSb_3$ and $IrSb_3$.

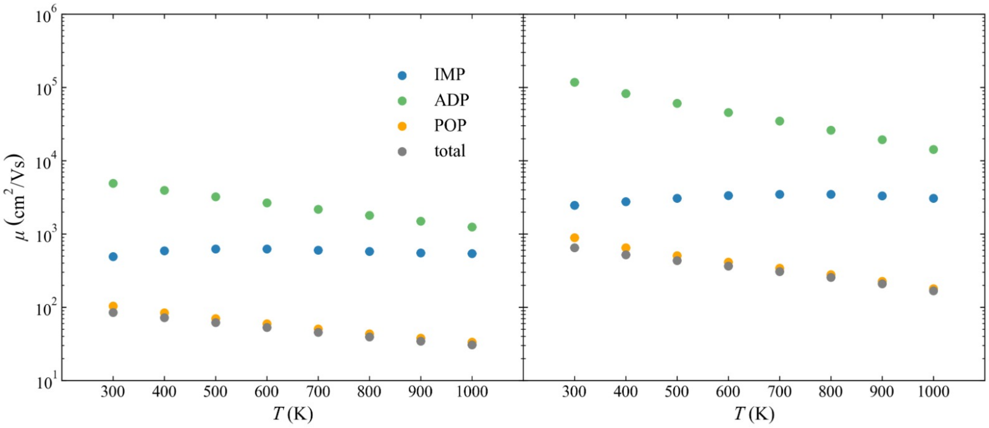

Figure S6: Total carrier mobility (black) and each scattering mechanism contribution for n-type (left) and p-type (right) $CoSb_3$ at $n = 10^{19}$ cm^{-3}
